# Clinical observations and management of a severe equine herpesvirus type 1 outbreak with abortion and encephalomyelitis

**DOI:** 10.1186/1751-0147-55-19

**Published:** 2013-03-05

**Authors:** Jasmin Walter, Christoph Seeh, Kerstin Fey, Ulrich Bleul, Nikolaus Osterrieder

**Affiliations:** 1 Klinik für Reproduktionsmedizin, Vetsuisse-Fakultät Universität Zürich, Winterthurerstrasse 260, Zurich, 8057, Switzerland; 2 Pferdegesundheitsdienst, Tierseuchenkasse Baden-Württemberg, Schaflandstrasse 3/3, Fellbach, 70736, Germany; 3 Klinik für Pferde – Innere Medizin, Justus-Liebig-Universität, Frankfurter Strasse 126, Giessen, 35392, Germany; 4 Institut für Virologie, Freie Universität Berlin, Philippstrasse 13, Berlin, 10115, Germany

**Keywords:** Equine herpesvirus type 1, Neuropathogenicity, Stud, Treatment, Recovery rate, Pregnancy rate

## Abstract

Latent equine herpesvirus type 1 (EHV-1) infection is common in horse populations worldwide and estimated to reach a prevalence nearing 90% in some areas. The virus causes acute outbreaks of disease that are characterized by abortion and sporadic cases of myeloencephalopathy (EHM), both severe threats to equine facilities. Different strains vary in their abortigenic and neuropathogenic potential and the simultaneous occurrence of EHM and abortion is rare. In this report, we present clinical observations collected during an EHV-1 outbreak caused by a so-called “neuropathogenic” EHV-1 G_2254_/D_752_ polymerase (Pol) variant, which has become more prevalent in recent years and is less frequently associated with abortions. In this outbreak with 61 clinically affected horses, 6/7 pregnant mares aborted and 8 horses developed EHM. Three abortions occurred after development of EHM symptoms. Virus detection was performed by nested PCR targeting gB from nasal swabs (11 positive), blood serum (6 positive) and peripheral blood mononuclear cells (9 positive) of a total of 42 horses sampled. All 6 fetuses tested positive for EHV-1 by PCR and 4 by virus isolation. Paired serum neutralization test (SNT) on day 12 and 28 after the index case showed a significant (≥ 4-fold) increase in twelve horses (n = 42; 28.6%). This outbreak with abortions and EHM cases on a single equine facility provided a unique opportunity for the documentation of clinical disease progression as well as diagnostic procedures.

## Background

Equine herpesvirus type 1 (EHV-1) is ubiquitous in horse populations worldwide. Many horses are latently infected with EHV-1 and reactivation of the virus can occur under stress, upon which latently infected carriers start to shed infectious virus that may spread to in-contact horses [1,2]. Clinical EHV-1 infection can manifest itself in the form of three different clinical syndromes: respiratory disease, usually mild, in horses under 2 years of age; abortions, typically in the last trimester of pregnancy; and equine herpesvirus myeloencephalopathy (EHM) [1-3]. Different strains vary in their abortigenic potential [4,5] as well as in neuropathogenicity [6]. A single nucleotide polymorphism (SNP) in the viral DNA polymerase (Pol) gene (ORF30) is considered one major marker for the neuropathogenic potential of EHV-1 strains [7,8]. The A_2254_/N_752_ and G_2254_/D_752_ Pol variants apparently differ in their capability of causing EHM [6,8,9]. A_2254_/N_752_ (non-neuropathogenic) viruses are mainly isolated from cases of abortion and less frequently from horses with EHM. On the other hand, the G_2254_/D_752_ Pol variant (neuropathogenic) is found predominantly associated with EHM outbreaks [10,11]. The estimated prevalence of latent EHV-1 infection varies between 54% and 88%, depending on the population sampled and the method of virus detection used [3,12]. In the last decades, the prevalence of the G_2254_/D_752_ EHV-1 genotype in EHV-1 positive horses in the United States has apparently increased from 3.3% in the 1960s to 19.4% at the beginning of this century [13-15]. This may indicate a selective advantage of the neuropathogenic strain, which also could increase prevalence of EHM in the future.

Simultaneous latent infection in lymphoid tissues with both A_2254_/N_752_ and G_2254_/D_752_ EHV-1 genotypes was documented in some studies [12,16,17]. Parallel reactivation of both genotypes was not addressed in these studies. In contrast, no dual infection with A_2254_/N_752_ and G_2254_/D_752_ EHV-1 was detected in 419 fetal isolates [15]. However, 2 EDTA blood samples of febrile horses tested positive for both genotypes in another study on one farm (n = 23; 20 abortion outbreaks, 3 EHM outbreaks) with EHM cases [14].

Detection of the D_752_ Pol variant in outbreaks without EHM cases is rare and occurred in only 5% of the cases in a retrospective worldwide study [7]. In a 23-year retrospective analysis, 11% (19/176) of isolates carried the G_2254_/D_752_ allele, of which 84% (16/19) were collected from EHM cases and only 16% (3/19) from respiratory or abortion cases [14]. A combination of abortions with EHM was documented in 3 of the sampled farms [14]. In Argentina, 7% (4/54) of the abortion outbreaks were induced by the G_2254_/D_752_ variant, and in 2 of these simultaneous neurologic disease occurred [18]. Two outbreaks with abortion and EHM cases caused by the neuropathogenic strain were recently documented in Croatia with breed-dependent clinical signs: a high incidence of abortion (53.1%) was described in Lippizaner mares compared to a lower incidence (10%) in Quarter horses infected with the identical virus [6].

Various reports with clinical data are available for outbreaks either with neurological cases or abortion storms [19-22]. This case report documents for the first time the clinical data collected during an EHV-1 outbreak with an uncommon combination of abortion and EHM on a single facility in Germany.

### Case presentation

This report is an account of an acute outbreak of EHV-1 in a mixed horse operation. Consequently, all clinical decisions and laboratory diagnostic procedures were initiated during the course of the outbreak. The documented outbreak occurred on an equine riding and breeding facility in southern Germany in early 2009. The stud includes different areas for sport and breeding horses. Broodmares and breeding stallions were kept separate from the sports horses. Breeding stallions were attended by grooms, which did not enter the broodmare or sport horse facilities as required for a European Union accredited breeding facility. In the sports horse section, horses were brought onto the premise without quarantine prior to entering the facility.

**Figure 1:**
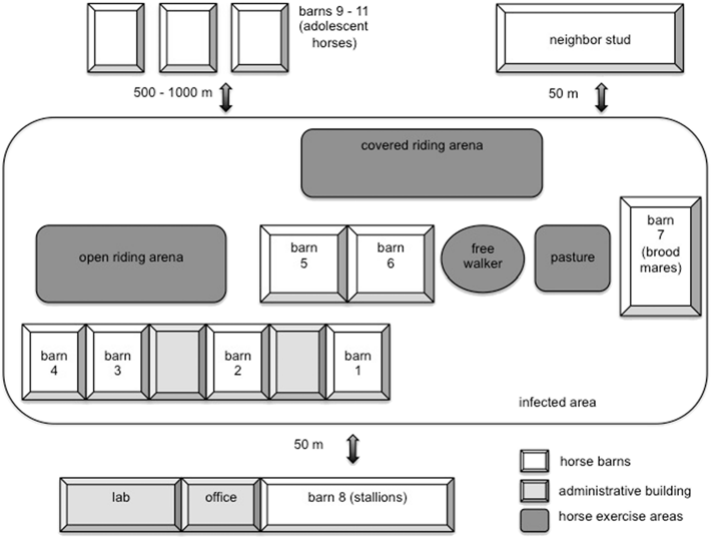
**Schematic presentation of the stud (not in scale).** A total of 61 clinically affected (fever, abortion, EHM) horses were recorded. For distribution of clinical cases in the barns see Table 1.

The clinical outbreak started in the broodmare section and spread rapidly to all other barns, except those for breeding stallions and adolescent horses. Affected horses were detected in seven of the eleven barns (Figure 1, Table 1). Adolescent horses (<2 years of age) were housed in groups of 20 in barns 9 to 11, where individual monitoring of body temperatures was not possible. In the affected areas, a total of 79 horses were housed. A stud in close proximity to the affected farm (50 m distance to broodmare barn 7) had no horses with fever, abortion or EHM, but no virus testing was performed. The vaccination status of the on-site animals was variable: All breeding stallions and broodmares were vaccinated^a^ in accordance with the manufacturers recommendations, with boosters in the 5^th^, 7^th^ and 9^th^ month of gestation. In barns 1 to 6, only 21 (32%) of the horses were vaccinated. The group of adolescent horses (barns 9 to 11) was not vaccinated. We view it as likely that the poor overall vaccination status contributed to rapid virus spread on the premise. Another contributing factor for the severity of the outbreak may be the typical boarding facility situation that includes frequent horse traffic on and off the premise, which may have compromised overall herd immunity.

**Table 1:** Description of horse populations in the different barns on the stud

**Barn**	**No. of horses**	**Affected horses**	**Horse description**^**a**^	**Same staff as horses of other barns**
1	6	5	SH; VH	yes
2	8	6	SH; VH	yes
3	12	8	SH; VH	yes
4	20	14	SH; VH	yes
5	12	10	SH; VH	yes
6	10	8	SH; VH	yes
7	11	10	BM; 1 foal	yes
8	10	0	BS	no
9-11	90	1 (?)^b^	AH	no

^a^SH = Sport Horses; VH = Visiting Horses; BM = Brood Mares; BS = Breeding Stallions; AH = Adolescent Horses.

^b^Clinical EHM signs, but no laboratory data available.

The index case was an abortion in barn 7. With the exception of the stallion barn and one questionable EHM case in the adolescent group (barns 9–11), all barns were affected. All affected barns were situated ≤ 50 meters next each other. The adolescent horses were stabled in barns 9–11, situated 500 to 1000 meters from the other barns. Horses from barns 1 – 7 used the same training facilities and paddocks.

The index case of the outbreak was an abortion in the broodmare barn 7. The day of the abortion is referred to as day 1 of the outbreak. Sixty-one clinical cases (77.2%) were detected during the outbreak and cases were identified over a period of 44 days. Onset, type and duration of clinical signs were documented for all individuals and are documented in the following sections.

#### Fever

In 71 horses, housed in barns 1 to 7, body temperatures were taken twice daily, starting on day 5 after the first abortion (index case). In the remaining 8 horses of the stabled population, control of body temperature as a routine procedure was practically impossible. Fever was defined as a body temperature of 38.1°C or higher (≥ 100.6°F).

**Figure 2:**
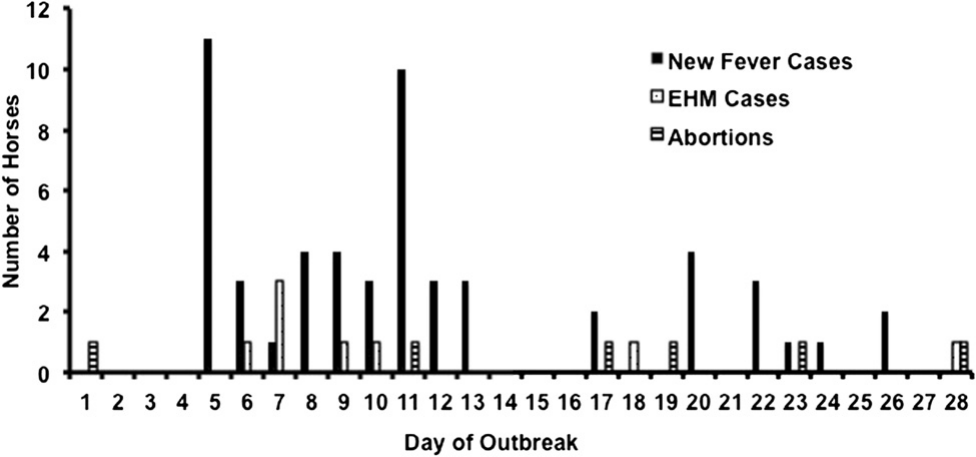
Number of newly detected clinical cases per day during the outbreak.

**Figure 3:**
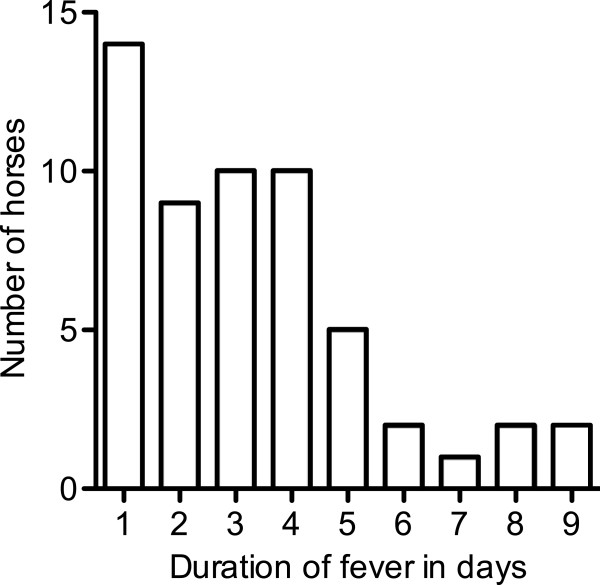
Duration of fever (≥38.1°C) in individual animals in days (n = 55/71).

An increase of body temperature was detected in 55 of the regularly measured 71 horses (77.5%). Since body temperature measurements had not started before day 5 of the outbreak, we assume that fever was missed in several horses in the beginning. The maximum number of fever cases/day was documented on day 5 with 11 horses. On day 11, a second peak with 10 new cases was observed (Figure 2). The mean of the horses’ maximal body temperatures was 39.1 ± 0.7°C (minimum 38.1°C, maximum 40.8°C) and a mean duration of 3.2 ± 2.2 days of fever were recorded (minimum 1 day, maximum 9 days, Figure 3). Seven horses (12.7%) were febrile for more than 5 days. Only 3 (6.5%) of the 55 horses showed a second febrile episode. Five horses with fever > 38.7°C with a duration of 5 to 8 days showed mild lethargy. Body temperatures of individual horses peaked mainly in the evening with normal temperature readings in the morning. Without the twice-daily assessment of body temperatures, many of the infected horses would have been missed, as fever was only recorded once in eleven horses. Without the “index” abortion or the first EHM cases, spread of the disease could have remained undiscovered, as most of the horses only having fever and no other symptoms remained in excellent general condition and did not develop obvious clinical signs. The regular recording of rectal temperature can be an effective diagnostic tool during an EHV-1 outbreak [11] and seems especially important when no respiratory signs were detected like during this outbreak.

#### Abortion

On day 1, 7 pregnant mares (8 to 11 months of gestation), including the index case mare, were housed in the broodmare barn 7. Six of the mares lost their foals (85.7%), one gave birth to a healthy foal. In four mares, the time between first fever and abortion ranged from 10 to 15 days (10, 10, 12 and 15 days) with fever durations of 3, 4, 2 and 4 days, respectively. In two mares that aborted on day 1 and 11, no fever was detected. Three of the mares developed EHM (Table 2) and abortions occurred 5, 7 and 10 days after the onset of neurological signs. One mare in the 8^th^ month of pregnancy had a premature placental separation (red bag delivery). One of the mares was also diagnosed with dystocia following the incorrect position of the dead foal and absence of uterine contractions. One EHV-1-positive foal was delivered alive 19 days before term. However, due to prematurity and distress at birth, it had to be euthanized for humane reasons. Four mares retained the fetal membranes after abortion (66.6%).

**Table 2:** Clinical and laboratory data in individual horses with EHM (n = 8) and/or abortion (n = 6)

**Animal**	**Ox**	**Ja**	**Ro**	**Wo**	**Le**	**Wa**	**Cl**	**Du**	**Ho**	**Da**	**Pu**
**Age/Gender**^**a**^	10/F	4/G	4/F	4/G	16/F	10/F	4/F	4/G	5/F	9/F	11/F
**Vaccination**	+	-	-	-	+	+	+	-	+	+	+
**Day of Onset of Fever/EHM/Abortion**^**b **^**(EHM grade)**^**c**^	-/-/1	-/9/- (2)	-/7/-(2)	-/7/-(2)	-/6/11 (4)	5/10/17 (4)	9/-/19	-/7/-(4)	13/-/23	13/18/28 (4)	20/28/-(5)
**Fever Max/Duration**^**d**^	-/-	-/-	-/-	-/-	-/-	39.2/2	39.4/3	38.9/3	39.1/4	39.8/4	39.4/8
**PCR**^**e **^**D12**	-/-/-	-/-/-	+/-/-	-/-/-	+/-/-	+/-/-	+/+/+	+/+/+	-/-/-	-/-/-	n.a.
**SNT**^**f **^**D12/D28**	384/192	64/48	32/64	32/128	128/128	48/96	16/128	128/192	48/128	96/128	n.a./96
**Fetus Cell Culture/PCR**^**g**^	+/+				+/+	-/+	-/+		+/+	+/+	

^a^gender: (F = female; G = gelding).

^b^given is the day of the outbreak.

^c^EHM grade according to Reed and Andrews (2004).

^d^Highest measured body temperature and duration of fever.

^e^PCR results from nasal swabs, serum and PBMC, separated by the dash.

^f^SNT values for the animals sampled on day 12 and 28 of the outbreak.

^g^Laboratory results of the fetus samples of the abortion cases (cell culture and PCR).

Animals are titled with name abbreviations. Ox is the index case. (n.a. = not available).

#### EHM

Eight of the 61 clinically affected horses (13.1%) developed neurological signs, which we all attributed to EHV-1 infection. Neurological symptoms were graded according to Reed and Andrews [23]. Fever was detected before onset of clinical signs of EHM in 3 of the 8 cases. In 5 horses, EHM symptoms became evident between day 5 and 9 of the outbreak without a preceding fever period, which could be the result of the delayed implementation of regular body temperature measurements. The incubation period for EHM symptoms in experimental and natural infected horses was previously described to range between 6 and 8 days [24]. In this outbreak EHM symptoms developed 5 (two horses) or 9 (one horse) days after the first fever, and 1, 2 and 4 days after the last fever (Table 2). As reported previously by other authors, all horses developing EHM were non-febrile at the onset of neurologic disease [19].

In three horses, neurological signs were restricted to mild ataxia of the hind limbs (grade 2). Four other horses suffered from more severe ataxia and upper motor neuron (UMN) symptoms as evidenced by an inability to open the urethral sphincter for autonomous urination (grade 4). One horse became temporarily recumbent (grade 5) [23]. The “UMN bladder” was observed between 2 and 13 days after onset of ataxia (2, 4, 10, 12 and 13 days in grade 4 and 5 horses). The average age of the eight EHM cases was 7.8 ± 4.5 years with a range of 4 to 16 years. In other studies, horses under five years of age had a decreased risk to develop EHM [19,20,22]. This outbreak included 4 EHM horses (50%) that were only 4 years old, with 3 of these younger horses developing grade 2 neurological signs. Just one horse with grade 4 neurological signs was 4 years old and the 3 other horses suffering from neurological signs grade 4 were over 9 years of age. Other authors also described a more severe clinical presentation in older horses. It was recently shown that the amount of virus in biological samples was not systematically related to the intensity of clinical signs observed [11]. During the outbreak described here, we too were unable to correlate virus detection and development of EHM (Table 2).

The 13.1% incidence of EHM observed in this outbreak appears relatively low. A cumulative analysis of records from the United States showed a 26% incidence of EHM in 452 EHV-1 infected horses, out of which 29% required euthanasia [25]. A recent report described a case fatality rate for EHV-1 of 71.4% (5 out of 7) [11]. The high mortality associated with EHM is at odds with our observations, where all 8 EHM cases survived and returned to their former level of activities, regardless of the severity of the disease. How the low EHM incidence and mortality were influenced by the implemented heparin treatment or supportive veterinary care remains speculative. Other reports document factors beside the neuropathogenicity of the virus (breed, sex, age or vaccination) that may contribute to the risk to develop severe EHM after EHV-1 infection and may be also responsible for the good outcomes in this outbreak [6,26,27].

#### Laboratory data

The laboratory results confirmed the clinical diagnosis “EHV-1 infection” that was based on the typical clinical course with fever (n = 55), abortions (n = 6) and neurological deficits (n = 8).

Gross pathology and histopathology were performed on all abortions. An ORF30 (Pol)-specific polymerase chain reaction (PCR) was used for detection of viral nucleic acid in fetal lung, liver and fetal membranes. Typical lesions in spleen and liver, with necrosis and intranuclear inclusion bodies, were detected in all 6 aborted fetuses. In 4 of the aborted fetuses, cell culture isolation of the virus was successful and in all 6 fetuses EHV-1-specific PCR amplification was positive. Sequencing of the amplification product was performed after digestion with restriction enzyme *Sal*I to identify the EHV-1 Pol D_752_ genotype [14].

**Figure 4:**
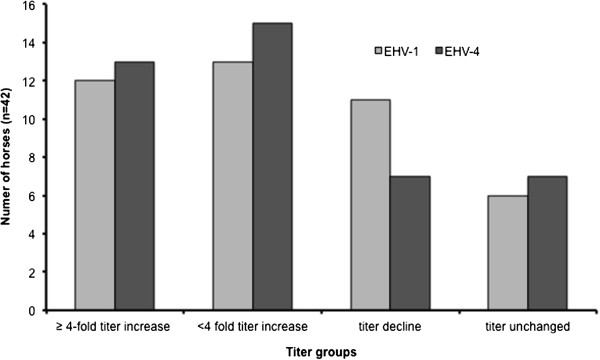
**Changes in EHV-1 and EHV-4 antibody titers of serum samples collected on days 12 and 28 after the index case.** A ≥ 4-fold increase in antibody titer is considered a significant rise. A simultaneous and significant increase of EHV-1 and EHV-4 antibodies was detected in 9 horses.

Due to practical reasons, on day 12 of the outbreak, nasal swabs, blood and sera were collected from 42 horses, irrespective of the development of clinical symptoms in individual horses. Antibody titer determinations specific for EHV-1 and EHV-4 were done using serum neutralization tests (SNT) and were repeated on day 28 of the outbreak [28]. A ≥ 4-fold titer increase was detected in 12 horses for EHV-1 (29%) and 13 horses for EHV-4 (31%). The highest EHV-1 antibody titer determined was 1:384 and was measured in the index mare on day 12. This low percentage of horses with seroconversion is probably the result of the delayed sampling, which was initiated only 12 days after the index case (Figure 4). Serum samples were analyzed in two batches on different days. Therefore, inter-test variability may have skewed the results. The rise in EHV-4 titers is likely due to the high cross-reactivity of antibodies against these closely related viruses [29]. These results confirm earlier reports suggesting SNT results are no reliable tool to diagnose acute EHV-1 infection, especially when the first sampling is not performed early in the beginning of disease in individual horses [30]. Virus detection in swabs, blood and peripheral blood mononuclear cells (PBMC) was performed by conventional nested PCR targeting glycoprotein B (gB) [31]. PCR diagnosed 11 EHV-1-positive nasal swabs, 6 positive sera and 9 positive PBMC samples; in total, 17 horses were positive for EHV-1 in at least one sample. All samples tested positive for EHV-1 in 3 horses. EHV-4 was not detectable in any of the samples. The results of our virological and serological tests confirmed that PCR of nasal swabs and PBMC samples should be initiated early in the febrile phase in suspect EHV-1 cases. Discrimination between EHV-1 and EHV-4 antibodies could have been achieved using an ELISA assay based on the C terminus of glycoprotein G [29]. But PCR and cell culture results from blood samples, nasal swabs and fetuses confirmed the clinical suspicion of an acute EHV-1 infection, which, in our opinion, decreased the importance of a differentiation of the antibody response. Additionally to routine diagnostic samples, semen samples of 3 infected non-breeding stallions, housed in the sport horse section, tested positive for EHV-1 by PCR, but not by virus isolation. Which role, if any, venereal shedding plays in EHV-1 spread requires further investigation [32].

#### Disease management

From day 1, quarantine was imposed for broodmare barn 7. From day 5, different grooms attended separate barns. Facilities were entered through hygienic barriers in which personnel had to change clothes. Horse movements were prohibited and all horses remained indoors. However, new cases of fever were detected in nearly all barns during the outbreak. We concluded from this observation that quarantine measures were implemented too late and infection had already been fully established in all areas of the premise (except the breeding stallion part). Separation of horses, which were suspected to shed virus based on clinical symptoms such as fever, EHM and/or abortion in the affected barns, was deemed impossible. Isolation facilities were not available and barn construction could not be changed fast enough, so horses were able to maintain nose-to-nose contact. All abortions occurred in the broodmare stall and aborted fetuses and placentas were submitted to pathological examination and taken with precautions in order not to contaminate the environment. The facility imposed on itself a voluntary quarantine and all horse transports on and off the stud were restricted from day 1.

#### Treatment

At the beginning of the outbreak, clinically healthy horses (n = 33) received prophylactic IM application of an immunostimulant based on inactivated parapoxvirus ovis (2 mL/horse; IM)^c^ given three times in two-day intervals. However, thirty-one (94%) of the treated horses developed a fever. Administration of a parapoxvirus ovis preparation was previously described as a successful measure to avoid severe clinical consequences of EHV-1 and -4 infection in young horses [33], but seemed to be not effective in our outbreak.

Horses showing fever as the only symptom received no other medication as long as they maintained good general condition and appetite. Three horses with fever were depressed and received flunixin-meglumine^d^ (1.1 mg/kg once daily for 1 – 3 days; IV). Mares that had an abortion also received flunixin-meglumine^d^ and were treated with heparin^b^ for laminitis prevention, benzathine-penicillin^e^ (10,000 I.U./kg every other day, three times; IM), gentamicin^f^ (4 mg/kg twice daily for 5 days; IV), oxytocin^g^ (15 I.U. as necessary; SC) and uterine lavage treatments.

In an attempt to prevent EHM, heparin^b^ (25.000 I.U.) was given twice daily for 3 days to 31 febrile horses from day 10 of the outbreak, while the first 30 horses exhibiting fever did not receive heparin. Horses showing signs of EHM were treated symptomatically [34]. The management was directed towards supportive nursing, nutritional care and reduction of CNS inflammation with non-steroidal anti-inflammatory drugs (flunixin-meglumine^d^,1.1 mg/kg once daily; IV). Generally, EHM horses showed good appetite but had to be fed at head level because uptake of food and water from the ground seemed to be difficult. EHM horses were also treated with flunixin-meglumine^d^, lactated Ringer solution and 5% glucose solution, depending on the horse’s general condition and laboratory results. Patients with UMN bladder were catheterized periodically as was deemed necessary. A combination of sulfamerazin/trimethoprim^h^ (45 mg/kg twice daily; PO) was administered for cystitis prevention until paralysis was resolved.

#### Disease Outcome

Horses with fever as the sole clinical sign remained in good general condition over the course of the outbreak and recovered without complication. Fertility of the aborting mares was good, as only 1 of the 6 aborting mares was barren in the following breeding season. All 8 EHM horses survived, ataxia and UMN bladder symptoms improved steadily and was resolved completely in all horses 3 months after infection.

## Conclusions

We document a clinical outbreak of EHV-1 caused by a G_2254_/D_752_ ORF30 variant virus resulting in 61 clinical cases. Infection caused fever (n = 55), abortion (n = 6) and/or EHM (n = 8) for 44 days in 7 barns. Strict containment procedures employed from day 5 of the outbreak did not prevent spread of virus on the premise. Abortion (n = 6) and EHM (n = 8) cases provided a unique opportunity to collect clinical data about disease progression and prognosis for recovery and fertility. Procedures to prevent EHV-1 outbreaks include in particular proper vaccination for newly introduced horses and the restriction of contact between brood mares, adolescent horses and sport horses with foreign contact [3,6,27].

## Consent

Written informed consent was obtained from the patients for publication of this report and any accompanying images.

### Endnotes

^a^Duvaxyn, Fort Dodge Animal Health

^b^Heparin-Natrium Braun “Multi”, Braun

^c^Zylexis, Pfizer Animal Health

^d^Paraflunixin, IDT

^e^Veracin-compositum, Albrecht

^f^Genta 100, CP-Pharma

^g^Oxytocin Albrecht, Albrecht

^h^Trimetox-Pulver, Veyx-Pharma GmbH.

## Abbreviations

EHM: equine herpesvirus-associated myeloencephalopathy; EHV-1: equine herpesvirus type 1; gB: glycoprotein B; IM: intramuscular; I.U.: international units; IV: intravenous; ORF: open-reading frame; PBMC: peripheral blood mononuclear cells; PCR: polymerase chain reaction; SNT: serum neutralization test; SC: subcutaneous; UMN: upper motor neuron

## Competing interest

The authors declare that they have no competing interests.

## Authors’ contributions

JW was the field veterinarian in charge of the stud, interpreted data and wrote the manuscript. CS was the responsible veterinarian of the Pferdegesundheitsdienst, Tierseuchenkasse Baden-Württemberg, an institution under public law, collected samples and interpreted results. KF gave advice for therapeutic interventions during the outbreak and drafted the manuscript critically. UB participated in data interpretation and writing of the manuscript. KO gave extensive advice on all aspects during the outbreak and contributed in drafting the manuscript. All authors read and approved the final manuscript.
